# The Role of Biomarkers in Influenza and COVID-19 Community-Acquired Pneumonia in Adults

**DOI:** 10.3390/antibiotics12010161

**Published:** 2023-01-12

**Authors:** Raquel Carbonell, Gerard Moreno, Ignacio Martín-Loeches, María Bodí, Alejandro Rodríguez

**Affiliations:** 1 Critical Care Department, Hospital Universitari Joan XXIII, 43005 Tarragona, Spain; 2 Department of Anaesthesia and Critical Care, St James’s University Hospital, Trinity Centre for Health Sciences, Multidisciplinary Intensive Care Research Organization (MICRO), D08 NHY1 Dublin, Ireland; 3 Critical Care Department, Hospital Universitari Joan XXIII, URV/IISPV/CIBERES, 43005 Tarragona, Spain

**Keywords:** biomarkers, procalcitonin, c-reactive protein, Influenza pneumonia, SARS-CoV-2, COVID-19, bacterial respiratory co-infection, prognosis

## Abstract

Pneumonia is a growing problem worldwide and remains an important cause of morbidity, hospitalizations, intensive care unit admission and mortality. Viruses are the causative agents in almost a fourth of cases of community-acquired pneumonia (CAP) in adults, with an important representation of influenza virus and SARS-CoV-2 pneumonia. Moreover, mixed viral and bacterial pneumonia is common and a risk factor for severity of disease. It is critical for clinicians the early identification of the pathogen causing infection to avoid inappropriate antibiotics, as well as to predict clinical outcomes. It has been extensively reported that biomarkers could be useful for these purposes. This review describe current evidence and provide recommendations about the use of biomarkers in influenza and SARS-CoV-2 pneumonia, focusing mainly on procalcitonin (PCT) and C-reactive protein (CRP). Evidence was based on a qualitative analysis of the available scientific literature (meta-analyses, randomized controlled trials, observational studies and clinical guidelines). Both PCT and CRP levels provide valuable information about the prognosis of influenza and SARS-CoV-2 pneumonia. Additionally, PCT levels, considered along with other clinical, radiological and laboratory data, are useful for early diagnosis of mixed viral and bacterial CAP, allowing the proper management of the disease and adequate antibiotics prescription. The authors propose a practical PCT algorithm for clinical decision-making to guide antibiotic initiation in cases of influenza and SARS-CoV-2 pneumonia. Further well-design studies are needed to validate PCT algorithm among these patients and to confirm whether other biomarkers are indeed useful as diagnostic or prognostic tools in viral pneumonia.

## 1. Introduction

Pneumonia is a main cause of morbidity, hospitalizations and health care costs worldwide [1,2,3], with a very high associated mortality rate, representing the fourth leading cause of global death in 2019, even before SARS-CoV2 pandemic, according to World Health Organization (WHO) estimates [4]. This low respiratory tract infection is classified into community-acquired pneumonia (CAP) and hospital-acquired pneumonia (HAP), and it can be caused by several infectious agents such as bacteria, viruses, fungi and parasites. In adults, bacteria are the most frequent microorganisms responsible of lung infection, hence bacterial CAP has been more extensively investigated. However, viruses are also common organisms causing pneumonia among adults, involved in up to 23% of CAP [5] depending on seasonal variations. Risk factors to develop viral pneumonia are immunosuppression, obesity, elderly patients and children, living in a long-term nursing care or chronic lung disease [6].

Over the last decades, the real incidence of viral pneumonia would probably be underestimated because available diagnostic tools, such as virus culture or viral antigen detection, did not have an adequate sensitivity. Advances in molecular biology techniques have revolutionized the procedures for detection and characterization of pathogenic viruses. Influenza virus, respiratory syncytial virus, rhinoviruses and coronavirus have been identified as the most common agents of viral pneumonia [7]. Moreover, respiratory viruses have become more relevant in recent years since the 2009 influenza A(H1N1) and the 2019 SARS-CoV-2 pandemics, which have re-emphasized the important role of respiratory viruses as causes of severe pneumonia, with a characteristic ease of transmission and a significant impact on mortality [8,9]. Likewise, viral pneumonia contributes substantially as a facilitator of bacterial infections (co-infections and super-infections). Viral pneumonia with bacterial co-infection is increasingly recognized as an underlying cause for CAP and HAP [7], which results in poorer patient outcomes with increased length of stay and mortality incidences [10,11].

In hospitalized patients with pneumonia, clinicians should make a conscientious effort to identify the causative pathogens of pneumonia to ensure an early and appropriate antibiotic prescription [12]. In the setting of viral pneumonia, the indiscriminate use of antibiotics should be avoided to mitigate the risks arising from an inadequate antimicrobial treatment, as it results in bacterial resistance, higher medical costs and risk of antibiotic-related adverse events. In fact, in the recent SARS-CoV-2 pandemic, high rates of inappropriate antibiotic up to 82% were prescribed in patients with viral pneumonia, in whom the incidence of bacterial co-infection was very low (8%) [13]. CAP is usually characterized by fever, increased sputum production, leukocytosis together with a newly recognized lung infiltrate on chest imaging. However, diagnosing pneumonia can be challenging. Clinical features that help to distinguish bacterial rather than viral causes of CAP are an hyperacute presentation, rapid onset of symptoms, presence of septic shock, leukocytosis with increased band forms, and dense segmental or lobar consolidation in chest radiograph [14]. Conversely, some findings raise suspicions of viral pneumonia, such as gradual symptom onset, lack of purulent sputum, other symptoms such as rhinitis, conjunctivitis or myalgia, previous exposure to infected contacts, and patchy or diffuse interstitial bilateral infiltrates in the chest radiograph [7,15]. Nonetheless, early identification of the pathogen causing pneumonia is difficult in clinical practice because signs and symptoms of viral and bacterial pneumonia are highly variable and often overlap, besides co-infections are common. From this perspective, biomarkers could be a useful tool for etiological diagnosis of CAP, to monitor the infection response to antibiotics and for prognosis, as well.

Procalcitonin (PCT) is a biomarker generally elevated in bacterial infections but not in viral, thus, it could be helpful for differentiating those respiratory tract infections caused by bacteria which would benefit from early antibiotic therapy [16]. Moreover, several studies have demonstrated a correlation between PCT and C-Reactive protein (CRP) with the severity of CAP [17,18]. The current scientific evidence concerning the role of biomarkers in viral pneumonia is more limited compared with the wide research of such biomarkers in bacterial CAP, and it is mainly based on studies conducted in influenza or SARS-CoV-2 pneumonia [19,20]. This review focuses mainly on PCT and CRP, since they are the most studied and most used biomarkers in daily clinical practice in the management of patients with pneumonia, given their clinical utility, low cost, and easy availability elsewhere.

The aim of the present investigation is to review the available scientific evidence about the role of biomarkers of infection for diagnosis, predicting etiology and prognosis of Influenza and SARS-CoV-2 pneumonia and those complicated cases of mixed viral and bacterial respiratory co-infection. To address the aim of this qualitative review, the authors performed a literature search using Pubmed/MEDLINE databases using the terms: “Biomarkers”, “Procalcitonin” and/or “C-Reactive Protein”, in combination with search terms including “Influenza Pneumonia”, “SARS-CoV-2 pneumonia”, “COVID-19”, “Bacterial co-infection”, “Mixed viral bacterial co-infection”, “Antimicrobial stewardship”, “Mortality”, “Prognosis”.

## 2. Procalcitonin and C-Reactive Protein as Biomarkers of Infection

Diagnosis of pneumonia is not always an easy practice since different respiratory diseases such as pulmonary hemorrhage, pulmonary edema, malignant or interstitial lung diseases can have a similar clinical presentation [21]. The specific confirmed diagnosis of pneumonia requires isolation of the pathogen in a culture but, in early stages of the infection, physicians base their diagnostic suspicion on data from the physical examination, vital signs, radiological studies and laboratory testing.

In light of this, inflammatory biomarkers have been broadly studied as useful complementary tools for identification of sepsis and particularly have been widely investigated in pneumonia. A biomarker is defined as “a characteristic that is objectively measured and evaluated as an indicator of normal biological processes, pathogenic processes, or pharmacologic responses to a therapeutic intervention” [22]. The ideal biomarker does not exist, but it should be helpful for early diagnosis of the infection, for monitoring the clinical response to an intervention and as a prognostic indicator of the disease. In the infectious disease context, the most routinely used biomarkers in clinical practice and also the most commonly studied in pneumonia are PCT and CRP. The main characteristics of both biomarkers are summarized in Table 1.

### 2.1. Procalcitonin: Physiology and Kinetics

PCT is the most common used biomarker in pneumonia due to its potential to differentiate bacterial infections from viral infections and non-infectious inflammatory diseases [23]. However, some factors different from bacterial infections may have an influence on PCT production, hence it is essential to understand the process of synthesis and release of PCT in order to make an adequate interpretation of plasma PCT levels in our patients.

PCT is composed of 116 amino acids and synthesized from the CALC-I gene located on chromosome 11. PCT is the precursor of the hormone calcitonin, which is involved with calcium homeostasis. In healthy individuals, it is synthesized in parafollicular cells (C cells) of the thyroid gland and in neuroendocrine cells of the lung and the intestine. The majority of PCT is subsequently processed by enzymatic proteolysis into three smaller peptides: the hormone calcitonin, katacalcin and an N-terminal fragment. Then, calcitonin is released into the plasma, and only a small quantity of PCT is secreted to the circulation, resulting in very low PCT levels (<0.1 ng/mL) under normal physiological conditions. The firsts reports about PCT in the medical literature were published in 1975 [24]. In 1993 Assicot et al. [25] reported for the first time that PCT levels were increased in septic patients. Based on several subsequent studies, it is known nowadays that in situations of sepsis, PCT is synthesized by macrophages and monocytic cells in many other tissues and organs as spleen, liver, kidney, testicles, fat, intestine, muscle or brain [26], so its blood levels rise significantly.

When an infection occurs, the causative pathogen generates an inflammatory response leading to a massive release of pro-inflammatory and anti-inflammatory cytokines in plasma. In case of bacterial infection, specific proinflammatory cytokines such as lipopolysaccharide (LPS), interleukin-1beta (IL-1β), interleukin-6 (IL-6), and tumor necrosis factor-alpha (TNF-α) as well as microbial toxins (endotoxins) stimulate CALC-I gene expression, and then the release of PCT is increased from diverse parenchymal tissues previously described [27]. Furthermore, Gram negative bacterial infections induce higher PCT production compared to Gram positive infections. This is explained by the distinct pathways of activation of the inflammatory cascades with different pathogen associated molecular patterns (PAMP) which lead to different cytokine profiles, hence different PCT values [28]. In contrast, in viral infections, there is a minimum elevation of PCT levels. This is due to the fact that virus stimulate in host T helper lymphocytes the release of interferon–gamma (IFN-γ), which inhibits the synthesis of TNF-α and release of IL- 1β [29], both required for the production of PCT in tissues.

Despite the high specificity of PCT for bacterial infections, its main limitation is that it could increase under certain circumstances of sterile inflammation, such as sterile pancreatitis [30], after major surgery [31], severe trauma [32], in cardiogenic shock [33], cardio-pulmonary resuscitation [34], rhabdomyolysis due to the ischemia-reperfusion injury and some malignancies [35], among others. Many of these situations generate damage associated molecular pattern (DAMP) leading to the production of relevant cytokines and, consequently, inducing an increase in PCT levels. In addition, PCT levels can be higher than normal baseline levels in patients with chronic kidney disease and PCT levels can significantly drop after continuous veno-venous hemodialysis [36]. The kinetics of PCT have been widely studied. After bacterial infection, PCT levels rise rapidly being detected within 2–3 h with a peak at 6–12 h. PCT has a half-life of approximately 25–30 h [37]. PCT release is not affected by systemic steroids, unlike CRP [38].

### 2.2. C-Reactive Protein: Physiology and Kinetics

Human CRP was first identified in 1930 in patients with pneumococcal pneumonia [39]. In healthy subjects, the concentration of blood CRP is less than 5 mg/L. It is a pentameric protein synthesized mainly by hepatocytes, although other tissues such as smooth muscle can also synthesize CRP. Its production is induced by IL-6, and in a lesser proportion by IL-1 due to inflammation of infection [40]. The physiological role of CRP is to bind different structures of the surface of microbial or injured cells in order to activate defense mechanisms of organism, complement system, opsonization and phagocytosis [41]. CRP is a nonspecific acute phase reactant, elevated in a variety of pathologies [42] such as trauma, surgery, burns, oncological diseases and immunological-mediated diseases. Therefore, due to its poor specificity is not useful to accurately differentiate inflammation from infection.

Some particularities in the production of CRP must be taken into account. CRP is synthesized in the liver hence, in situations such as liver failure, hypoproteinemia or cirrhosis [43], CRP levels are lower. The use of systemic corticosteroids is also associated with lower increases in CRP levels because glucocorticoids inhibit many of the initial events in an inflammatory response, causing a reduction of multiple proinflammatory cytokines responsible for CRP release [38]. The rise in CRP is slower compared with PCT, and CRP levels could be detectable within 6–12 h and peaks within 36–50 h after the inflammation onset. CRP half-life is approximately around 19 h [44].

## 3. The Role of Procalcitonin and C-Reactive Protein in Identifying Influenza Pneumonia, SARS-CoV-2 Pneumonia and Mixed Bacterial and Viral Respiratory Co-Infection

Early differentiation between bacterial or viral etiology of CAP could lead to an adequate treatment of our patients as well as to a significant reduction in the administration of inappropriate antibiotics, which constitutes a globally public health problem. However, this could be challenging since bacterial and viral infections can cause similar signs, symptoms and radiological findings [7]. In the case of bacterial pneumonia, microbiological culture is the definitive method for diagnosing the infection. However, cultures require more than 24 h for confirmation. In addition, blood cultures, legionella tests, and pneumococcal urinary antigens are usually taken. The recent implementation of polymerase chain reaction (PCR) assays allows early detection of bacterial deoxyribonucleic acid (DNA) in respiratory samples, in spite of it is not extensively available in all health care institutions.

The laboratory diagnosis of viral pneumonia is more complex. It has been based on viral cultures, but are expensive, not widely available and do not allow the identification of all viruses. The rapid detection of viral antigens by immunofluorescence has also been proposed, however, this method has low sensitivity and the detection of serum antibodies requires a blood sample both in the acute phase and in the convalescent phase of the infection, thus it is not useful for the early diagnosis of viral infection. In recent years, the ability to early detect the presence of viruses in respiratory samples has been improved with the advance of genetic amplification techniques such as PCR or Reverse Transcriptase PCR, which constitute the most sensitive and specific techniques available to date for the identification of respiratory viruses. It must be emphasized that a potential drawback of molecular methods is the detection of nonviable organisms, hence the detection of viruses and/or bacteria DNA in the lower respiratory tract does not guarantee the causation of pneumonia.

The aforementioned strategies carry certain limitations for early detection both viral and bacterial respiratory tract infection. Therefore, it has been extensively studied that biomarkers could be of help in this diagnostic challenge.

### 3.1. Procalcitonin Diagnostic Ability

PCT is probably the most studied biomarker in the etiological diagnosis of pneumonia, given its early increase after infection of bacterial origin. The recommendations emanating from guidelines provided from American Thoracic Society (ATS) and Infectious Diseases Society of America (IDSA) for diagnosis and treatment of CAP in adults [45], stated that empiric antibiotic therapy should be initiated when CAP is suspected, regardless of initial serum PCT level. However, these guidelines base their recommendation on the results of different studies in which a PCT cut-off point alone fails to unequivocally differentiate bacterial pneumonia. Despite them, scientific evidence supports the use of PCT in the antibiotic stewardship among patients with CAP. Several studies have shown that PCT levels >0.25 ng/mL indicate a high likelihood of bacterial respiratory tract infection, whereas PCT levels <0.1 ng/mL pointed out that bacterial infection is unlikely and suggest that other causes of pneumonia should be expected [23,46]. Christ-Crain et al. [47], in a randomized control trial (RCT) of 302 patients with suspected CAP, evaluated the value of PCT for the initiation and duration of antibiotic therapy. In the control group, antibiotics were administered according to usual practice and in PCT group, antibiotic was not started or it was withdrawn when PCT levels were less than 0.25 ng/mL. They reported that initial empiric antibiotic therapy was appropriate in 97% of patients when it was started based on PCT levels, with a reduction of antibiotic administration in the PCT group. A large Cochrane review [46] of 14 RCT evaluating the feasibility of using PCT for starting and stopping antibiotics in different populations with acute respiratory infections (ARI), showed that PCT guidance was not associated with treatment failure or increased mortality in any clinical setting. These results were robust in various sensitivity analyses. Moreover, a recent update of the previous Cochrane review aimed to assess the safety of PCT-guided antibiotic stewardship [48], reported that the use of PCT to initiation of antibiotic treatment resulted in lower risk of mortality, lower antibiotic use, lower risk for antibiotic-related side effects, along with similar results for different clinical settings and types of ARI. Despite the fact that most of medical literature on the use of PCT in pneumonia is found in hospitalized patients, there is also scientific evidence supporting the use of PCT in outpatients. Schuetz et al., conducted a meta-analysis to assess the safety of PCT-guided treatment in patients with ARI from different clinical settings, including, among others, two primary care trials with 1008 patients, in which they observed a reduced antibiotic exposure and antibiotic side-effects in PCT-guided patients.

Not only the Cochrane organization, but also the Food and Drug Administration (FDA) [49] and the WHO [50] support the use of PCT to guide antibiotic initiation or discontinuation in patients with severe respiratory infections. The authors of the current review support the recommendation from these recognized organizations. We consider that, although there is no ideal cut-off point for PCT to identify bacterial respiratory infection, PCT levels sharply increase in its presence and remain low in most of purely viral or fungal infections. Moreover, the safety of PCT protocols to guide antibiotic initiation for the treatment of pneumonia has been widely demonstrated. Accordingly, we believe that this biomarker is highly useful for antibiotic stewardship in patients with severe pneumonia. It should be noted that PCT is more expensive than CRP, but cost-effective when the costs of antibiotic prescription and antibiotic resistance are considered [51].

Based on PCT synthesis process, it has been demonstrated that low PCT levels cannot accurately predict the viral etiology of pneumonia. Otherwise, bacterial super- or co-infections in patients with confirmed viral pneumonia facilitate the synthesis of PCT. Thus, PCT has also been proposed as a useful tool to rule out bacterial co-infection and, consequently, to avoid inappropriate antibiotics in these patients. Most studies have been conducted among patients with influenza and SARS-CoV-2 pneumonia to shed light on this issue.

Bacterial co-infections are common in influenza pneumonia ranging from 20 to 30% and are associated with worse outcomes [11]. Owing to the high incidence of bacterial co-infection in patients with influenza pneumonia and the fact that data regarding the ability of PCT to diagnose or to exclude co-infection is limited, recent clinical practice guidelines [45] recommended starting empiric antibiotics in adults with CAP who test positive for influenza, in both inpatient and outpatient settings.

Few retrospective studies have focused on the ability of PCT to identify bacterial respiratory co-infection in patients with influenza pneumonia, showing that different cut-off points are associated with low sensitivity and specificity for that purpose [52,53]. Nevertheless, in a large cohort of 1608 patients with severe influenza pneumonia, we reported that those with bacterial co-infection presented higher PCT levels (4.25 [0.6–19.5] versus 0.6 [0.2–2.3] ng/mL) compared to those with pure viral pneumonia [53]. Otherwise, meta-analyses and studies focused on the ability of PCT to rule out bacterial respiratory co-infection in patients with influenza pneumonia have found favorable results based on its good negative likelihood ratio that support the use of PCT [54]. Rodríguez et al. [55], performed an observational study including 972 critically ill patients with Influenza A(H1N1) pneumonia (20% with bacterial co-infection) using Chi-squared Automatic Interaction Detection (CHAID) analysis and revealed that PCT level on ICU admission <0.29 ng/mL showed a sensitivity of 88.2%, and high negative predictive value (NPV) of 91.9% to rule out bacterial co-infection, improving to NPV of 94% in absence of shock. Cuquemelle et al. [56], in a multicenter retrospective observational study conducted in 23 French ICUs involving 103 patients with confirmed Influenza A(H1N1) pneumonia, showed that a cutoff of PCT on admission of >0.8 ng/mL, the sensitivity and specificity for distinguishing mixed viral and bacterial pneumonia were 91 and 68%, respectively. Besides, a PCT level <0.8 ng/mL combined with the lack of alveolar condensation was strongly associated with the absence of bacterial co-infection (OR 12.9, 95% CI 3.2–51.5; *p* < 0.001).

Since the beginning of the SARS-CoV-2 pandemic, a large number of studies have assessed the ability of PCT for diagnosing bacterial co-infection in COVID-19 patients. Unlike influenza, the incidence of bacterial co-infection associated with COVID-19 is much lower than initially expected, around 8% [57]. These cases of mixed viral and bacterial pneumonia have been associated, as in influenza pneumonia, with worse outcomes and higher mortality [58]. Therefore, early identification of bacterial co-infection and optimal appropriate antibiotic treatment is crucial among COVID-19 patients. Similar to influenza infection, several studies failed to demonstrate that high PCT levels are good predictors of bacterial respiratory co-infection in patients with SARS-CoV-2 pneumonia [59,60,61]. Nevertheless, different studies have identified a high NPV of PCT to rule out bacterial respiratory co-infection. Dolci et al. [62] observed that a PCT cutoff <0.25 ng/mL held a high NPV of 91.7% to exclude bacterial co-infection. May et al. [61] also reported that a PCT threshold of <0.25 ng/mL had a NPV of 99% for ruling out bacterial respiratory co-infection. Similar results have been found for our working group [59] in a large multicenter study including 4635 critically ill patients with SARS CoV-2 pneumonia We found that a PCT threshold of <0.3 ng/mL could be helpful to rule out bacterial respiratory co-infection on ICU admission, with a NPV of 91.1%.

**Figure 1:**
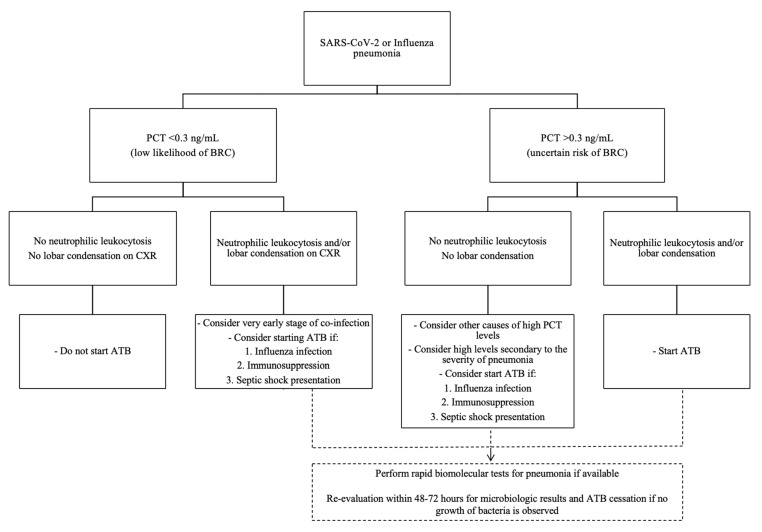
Procalcitonin-based algorithm for the decision to initiate, continue or stop antibiotics for patients with Influenza or SARS-CoV-2 pneumonia. Abbreviations: PCT: procalcitonin; BRC: bacterial respiratory co-infection; CXR: chest X ray; ATB: antibiotic.

Recognizing bacterial co-infection among patients with SARS-CoV-2 and Influenza pneumonia requires a high index of suspicion. Despite this challenging diagnosis, it is not justified to start antibiotics indiscriminately to all patients with viral pneumonia. Some features of the presence of associated bacterial respiratory co-infection may still be identifiable despite a significant overlap of viral and bacterial infection. Neutrophilic leukocytosis is the hallmark of bacterial pneumonia, along with lobar or segmental air-space opacification with air bronchograms [63]. However, in severe viral respiratory infections these characteristics may be present in patients who develop critical illness with acute respiratory failure. Hence, we are convinced that initial values of PCT may help clinicians with the decision to initiate or withdrawn antibiotic treatment so as to avoid overprescribing antibiotics when unnecessary. Based on the aforementioned scientific evidence and previously recognized associated factors with bacterial co-infection in viral pneumonia as immunosuppression or septic shock presentation [60,64,65] we propose a practical PCT algorithm for clinical decision making to guide antibiotic initiation in cases of Influenza and SARS-CoV-2 pneumonia (Figure 1). Likewise, in case of not prescription of antibiotics in suspected viral CAP based on a medical decision supported by low PCT levels, it would be recommended a close monitoring and follow up, reconsidering to administer antibiotic treatment if rapid clinical deterioration or promptly increase of PCT levels within 24–48 h.

### 3.2. C-Reactive Protein Diagnostic Ability

The potential of CRP to identify the etiology of pneumonia has been extensively investigated. Based on its physiology, it is an acute phase protein synthesized mainly in response to IL-6 which is released in multiple and very diverse situations of inflammation and pathological processes, thereby levels of CRP do not specifically increase depending on the type of microorganism that causes the infection.

The current scientific evidence derived from previous observational studies and systematic review and meta-analysis [66] do not support the use of CRP to guide antibiotics prescription, due to its scarce sensitivity and specificity for detection of viral or bacterial lower respiratory tract infection [64]. Consistently, international guidelines did not mention regarding the use of CRP values for the diagnosis of CAP [45]. Moreover, our working group observed that CRP was not a useful biomarker to identify bacterial co-infection neither in influenza nor in COVID-19 pneumonia [53,59]

## 4. The Prognostic Value of Procalcitonin and C-Reactive Protein in Influenza and SARS-CoV-2 Pneumonia

### 4.1. PCT as Severity Predictor

The ability to reliably evaluate clinical severity and predict worse outcomes and treatment failure is essential in patients with pneumonia. In bacterial CAP, current evidence confirms that PCT is useful to assess the prognosis of the disease [65,67]. Moreover, the increase in PCT levels correlates closely with the host’s inflammatory response to infection, and its plasma concentration declines meanwhile patients improve clinically from the bacterial infection. Therefore, in bacterial pneumonia, daily monitoring of PCT levels is an effective and safety tool to guide the duration and discontinuation of antimicrobial treatment [68,69].

Minor evidence exists on the prognostic role of PCT in viral pneumonia. However, the available scientific research to date is in line with the significant prognostic role of PCT in viral CAP. Gautam et al. [60], analyzed 2075 patients with viral infection, showing that PCT raised in proportion to disease severity and it was not suppressed by interferon signaling, in contrast to prior models of PCT regulation. Cytokines such as IL-1β, IL-6 and TNF-α could play a significant role in the mechanism of severe viral pneumonia driving PCT production, without influenced by IFN-γ in the absence of bacterial co-infection. Otherwise, viruses could stimulate the expression of PCT through mechanisms independent of cytokines, although further well-designed confirming studies are required to elucidate these putative molecular pathways.

Several studies have also demonstrated that severe influenza H1N1 pneumonia is associated with elevated PCT levels, in the absence of proven bacterial co-infection [70,71]. Interestingly, similar findings have been recently found in SARS-CoV-2 pneumonia. Lippi et al. [72] performed a meta-analysis carried out in the early stages of the pandemic and reported that elevated PCT values were associated with a four-fold increase risk of severe SARS-CoV-2 infection. The vast majority of meta-analyses conducted agree that augmented PCT levels are significantly associated with higher mortality [19,73]. Our working group [59], evaluated 4635 COVID-19 critically ill patients in a multicenter study and found that PCT was a biomarker significantly associated with ICU mortality (OR 1.5, CI 95% 1.18–1.84; *p* < 0.001). Similar findings have been observed in several studies reporting the association of elevated PCT concentrations with worse outcomes, even with higher mortality regardless the presence of bacterial co-infection [74,75,76].

Definitely, although it has been faithfully believed that PCT values might be useful to identify the presence of bacterial co-infection in patients with viral pneumonia, its measurements only have certain ability in ruling out bacterial co-infection, besides high levels reflect the severity of viral respiratory infection regardless the possibility of bacterial co-infection, if present.

### 4.2. CRP as Severity Predictor

CRP is a well-stablished prognostic marker in CAP [17,77]. It has been found to be an independent predictor of stability, as low levels of CRP in addition to clinical situation might improve the prediction of absence of severe complication in CAP [78]. Nevertheless, due to its low specificity, monitoring the daily trends of CRP concentration is less useful to guide antimicrobial treatment, as it may be related to other noninfectious inflammatory disorders [79].

Regarding viral pneumonia, some data have shown an association of higher levels of CRP with the severity of the infection. In influenza pneumonia few studies have been designed with the aim of evaluating the prognostic value of CRP [53,80]. There has been more clinical research in SARS-CoV-2 pneumonia, in which data from meta-analyses confirmed that CRP values were also associated with higher disease severity [53,59].

## 5. Other Biomarkers in Influenza and SARS-CoV-2 Pneumonia

There are many other promising biomarkers with important value in CAP, but their role in viral pneumonia has been poorly studied.

Midregional-proadrenomedullin (MR-proADM) is not useful for differentiating bacterial from viral pneumonia, but its levels have a significant prognostic role in both bacterial and viral pneumonia. It has been reported in hospitalized CAP patients that MR-proADM is better predictor for both short and long-term mortality, than other biomarkers such white blood cells, CRP or PCT [81,82]. In influenza pneumonia, different prospective, observational studies have confirmed the prognostic value of MR-proADM [83,84]. Similarly, a recent systematic review and meta-analysis confirms that MR- proADM is usefull to predict mortality among the critical COVID-19 population [85].

Presepsin is a fragment of monocyte lipopolysaccharide CD14 receptor that is released during monocyte-macrophage activation in response to infectious pathogens. It is not an specific biomarker for diagnosis of pneumonia nor of its microbial etiology, but several clinical studies have demonstrated that presepsin is a reliable tool to assess severity in viral pneumonia [86], with a similar role in SARS-CoV-2 pneumonia [87].

Soluble urokinase-type plasminogen activator receptor (suPAR) production is correlated with immune system activity and is a biomarker to indicate the severity and deterioration of many inflammatory diseases. The research on suPAR in the field of viral pneumonia is limited, its prognostic role has recently been investigated in SARS-CoV-2 pneumonia [88]. However, scientific clinical evidence is needed to confirm it.

Dimer-D is a product of fibrin degradation, widely used for the diagnosis of thromboembolic disease. Otherwise, Dimer-D levels have been shown to be related to the severity of influenza viral pneumonia [89] and SARS-CoV-2 pneumonia [90].

Given the limited medical literature available on this matter, further studies are needed to confirm whether previous biomarkers are indeed useful as diagnostic or prognostic tools in influenza and SARS-CoV-2 pneumonia in routine clinical practice.

## 6. Future Directions

Further research is required regarding improving diagnostic and management of patients with viral pneumonia. The search for better biomarkers with higher sensitivity and specificity than the existing for the diagnosis and management of viral CAP should continue.

Owing to the epidemiological evidence that Influenza and SARS-CoV-2 are a significant cause of CAP, there is an imperative need to validate the use of new rapid laboratory tests, to accurately distinguish the etiologic cause of pneumonia for a better antibiotic stewardship. Further well-design studies are needed to validate PCT algorithm-based antibiotic decision-making in patients with viral pneumonia.

## 7. Conclusions

This review updates the clinical value of the most widely used biomarkers in daily practice, PCT and CRP, in Influenza and SARS-CoV-2 pneumonia. Based on the scientific evidence, CRP is not able to diagnose viral or bacterial cause of pneumonia nor to identify cases of mixed viral with bacterial co-infection. In the case of PCT, it has been clearly demonstrated that high levels of this biomarker indicate a high likelihood of bacterial pneumonia. Therefore, PCT is a useful biomarker in conjunction with other clinical, radiological and laboratory data, to make the decision to start or not the empirical antibiotic treatment in these patients.

In patients with CAP, despite the advances in molecular techniques, the early distinction of the microorganism causing the infection remains a diagnostic challenge, resulting in an overuse of antibiotics worldwide with a subsequent increase in antibiotic resistance, which constitutes an important global health problem that must be addressed. Consequently, physicians should make a conscientious effort to guide correct treatment. In this regard, among patients with influenza and SARS-CoV-2 pneumonia, PCT might be helpful to exclude bacterial respiratory co-infection in combination with clinical data, providing to clinicians a valuable tool to guide antibiotic stewardship.

Furthermore, both PCT and CRP are valuable prognostic markers with significant association with clinical outcomes and mortality in influenza and SARS-CoV-2 pneumonia. The decision for or against the use of antibiotics always remains a clinical decision, but a correct use of PCT with proper training of physicians and considering its limitations, could improve the management of pneumonia, helping to mitigate the global bacterial resistance crisis.

## Figures and Tables

**Table 1:** Main characteristics of PCT and CRP production, kinetics and its main role in pneumonia.

Biomarker of Infection	Procalcitonin	C-Reactive Protein
Production organ	Thyroid gland, lung, spleen, liver, kidney, fat, intestine, muscle, brain	Liver
Infective stimulators factors	IL-1β, IL-6, TNF-α, LPS, microbial endotoxins (bacteria)	IL-6, IL-1 (infections)
Infective Inhibitors factors	IFN-γ (virus and fungi)	
Non-infective causes of biomarker production	Pancreatitis, surgery, severe trauma, cardiogenic shock, cardio-pulmonary resuscitation, certain malignancies, cytokine storms	Any systemic inflammation: trauma, surgery, burns, certain malignancies, immunological-mediated inflammatory diseases…
Affected by	Kidney disease, CVVH	Systemic corticosteroids
Production time from infection (hours)	2–3	6–12
Half-life time (hours)	25–30	19
Peak time from infection (hours)	12	48
Main role in pneumonia	Exclude BRC in viral CAP, guide of duration of antibiotic, prognosis	Prognosis

Abbreviations: IL-1β: interleukin-1beta; IL-6: interleukin-6; TNF-α: tumor necrosis factor-alpha; LPS: lipopolysaccharide; IFN-γ: interferon–gamma; CVVH: continuous veno-venous hemodialysis; BRC: bacterial respiratory co-infection; CAP: community-acquired pneumonia.

## Data Availability

Not applicable.
